# Association of HLA alleles with *Plasmodium falciparum* severity in Malian children

**DOI:** 10.1111/j.1399-0039.2011.01661.x

**Published:** 2011-06

**Authors:** K E Lyke, M A Fernández-Viňa, K Cao, J Hollenbach, D Coulibaly, A K Kone, A Guindo, L A Burdett, R J Hartzman, A R Wahl, W H Hildebrand, O K Doumbo, C V Plowe, M B Sztein

**Affiliations:** 1 Department of Medicine, Center for Vaccine Development, University of Maryland School of Medicine, Baltimore, MD, USA; 2 Laboratory Medicine, The University of Texas, MD Anderson Cancer Center, Houston, TX, USA; 3 Comprehensive Transplant Center, HLA Laboratory, Cedars-Sinai Health System, Los Angeles, CA, USA; 4 Department of Pediatrics, Georgetown University Medical Center, Washington, DC, USA; 5 Center for Genetics, Children's Hospital Oakland Research Institute, Oakland, CA, USA; 6 Malaria Research and Training Centre, Faculty of Medicine, Pharmacy and Dentistry, University of Bamako, Bamako, Mali; 7 Division of Cancer Epidemiology and Genetics, National Cancer Institute, National Institutes of Health, Bethesda, MD, USA; 8 Core Genotyping Facility, SAIC-Frederick, Inc., NCI-Frederick, Frederick, MD, USA; 9 C.W. Bill Young DoD Marrow Donor Program, Naval Medical Research Institute/Georgetown University, Kensington, MD, USA; 10 Department of Microbiology and Immunology, University of Oklahoma Health Sciences Center, Oklahoma City, OK, USA; 11 Center for Vaccine Development, Howard Hughes Medical Institute, University of Maryland School of Medicine, Baltimore, MD, USA

**Keywords:** children, human leukocyte antigen class I, human leukocyte antigen class II, malaria, Mali, *Plasmodium falciparum*

## Abstract

Pre-erythrocytic immunity to *Plasmodium falciparum* malaria is likely to be mediated by T-cell recognition of malaria epitopes presented on infected host cells via class I and II major histocompatibility complex (MHC) antigens. To test for associations of human leukocyte antigen (HLA) alleles with disease severity, we performed high-resolution typing of HLA class I and II loci and compared the distributions of alleles of HLA-A, -B, -C and -DRB1 loci in 359 Malian children of Dogon ethnicity with uncomplicated or severe malaria. We observed that alleles A*30:01 and A*33:01 had higher frequency in the group of patients with cerebral disease compared to patients with uncomplicated disease [A*30:01: gf = 0.2031 *vs* gf = 0.1064, odds ratio (OR) = 3.17, *P* = 0.004, confidence interval (CI) (1.94–5.19)] and [A*33:01: gf = 0.0781 *vs* gf = 0.0266, 4.21, *P* = 0.005, CI (1.89–9.84)], respectively. The A*30:01 and A*33:01 alleles share some sequence motifs and A*30:01 appears to have a unique peptide binding repertoire compared to other A*30 group alleles. Computer algorithms predicted malaria peptides with strong binding affinity for HLA-A*30:01 and HLA-A*33:01 but not to closely related alleles. In conclusion, we identified A*30:01 and A*33:01 as potential susceptibility factors for cerebral malaria, providing further evidence that polymorphism of *MHC* genes results in altered malaria susceptibility.

## Introduction

Malaria's strong selective force upon the human genome is evidenced by host adaptations such as the hemoglobinopathies (S, C and E), glucose-6-phosphatase deficiency, Duffy-negative blood groups and α^+^-thalassemia, all of which limit malaria disease and mortality 1–6. Genetically regulated immune responses to malaria may, similarly, be influenced by gene polymorphisms in class I and II major histocompatibility complex (MHC) antigens. African populations present the largest genetic diversity in the human leukocyte antigen (HLA) system. Within class I loci alone, extensive allelic diversity has been documented with more than 600 HLA-A, 1100 HLA-B and 400 HLA-C alleles defined by variations at the protein sequence level (7). This diverse genetic repertoire reflects the long divergence time of African populations in a setting with numerous environmental pathogens. The Dogon ethnic group in Mali, who live near the ancient sub-Saharan trade center of Timbuktu, represents a unique African ethnicity with particularly high allelic diversity and genetic distances from ancestral African populations such as the Kenyan Luo population (7). This genetic diversity may pose a significant obstacle to the development of malaria vaccines aimed at eliciting cell-mediated immunity (CMI).

The impact of genetic polymorphism upon the human immune response is recognized but poorly understood. The importance of HLA haplotypes in protection from natural infection was first indirectly shown by the association of HLA-B53 and the HLA class II block DRB1*13:02-DQB1*05:01 with protection against severe malaria in West Africa (8). Cytotoxic T-cell responses to conserved epitopes of liver stage antigens-1 and -3 (LSA-1 and LSA-3) restricted by the class I allele B53 have been shown 9, 10. T-helper (Th) cell responses to epitopes within the circumsporozoite protein (CSP) and LSA-1 restricted by class II alleles have been shown to protect against severe malaria (11), malaria-associated anemia and reinfection (12). Regional variations in the distribution of HLA alleles complicate the approach to understanding immune correlates of protection 12–14. Moreover, genetic variations can also cause a predisposition to variations of malarial disease such as severe malaria (15) and cerebral malaria (13). The HLA–peptide complexes generated following exposure to a stimulatory pathogen may influence T-cell production of Th1- and Th2-specific cytokine patterns. While this is not a clearly established phenomenon, interferon-γ (IFN-γ) and interleukin (IL)-10 production has been shown to be associated with specific HLA polymorphisms after rubella vaccination (16). Malaria severity and subsets of malarial disease show discrete cytokine secretion patterns (17) but, to our knowledge, no relationship has yet been established between HLA polymorphisms and cytokine production in diverse clinical malaria presentations.

To examine previously described associations and to investigate if other HLA factors play a differential predispositional role in the development of clinically distinct disease courses after infection with *Plasmodium falciparum,* we performed pair-wise group comparisons in a population of ethnically homogenous, Malian Dogon children with severe malaria who were age- and residence-matched to children with uncomplicated malaria. Differences in the distributions of alleles at the loci, A, B, C and DRB1 of the HLA system were assessed and the relationship between HLA polymorphisms and cytokine production was examined. Our results help define the association of defined HLA alleles with clinical outcomes as a result of the selection force of malaria on the human genome, and illustrate further the complexity of African genetic diversity as it relates to the development of malaria T-cell vaccines. This study also establishes an association between HLA polymorphisms and IL-10 production in severe malaria.

## Materials and methods

### Study design and enrollment

Malian children aged 3 months to 14 years presenting with clinical symptoms consistent with malaria were enrolled into a matched case–control study evaluating the risk and protective factors for severe malaria. The study was conducted at the Bandiagara Malaria Project research clinic in Bandiagara, a rural town of 13,634 inhabitants in the Dogon country in northeast Mali. Malaria transmission is seasonal and heavy with children aged less than 10 years having an average of 2 (range 0–4) clinical malaria episodes per transmission season (18) and severe malaria affecting 2.3% of children less than 6 years of age every year (19). The malaria transmission season extends from July to December. The dominant self-reported ethnic group is Dogon (∼80%) with Peuhl, Bambara and other ethnic groups also present. Over the course of three malaria transmission seasons, from October 1999 to January 2003 (preceding clinical trial registry), 253 index cases of severe malaria from Bandiagara and surrounding areas were admitted to the Bandiagara Malaria clinic. Each index case was matched by age, ethnicity and residence to a case of uncomplicated malaria and a healthy control within 5 days of enrollment. For the purposes of HLA analysis, only those individuals self-identified as Dogon were examined. Full details of enrollment and case definitions are reported elsewhere 20, 21.

The trial was conducted in compliance with the International Conference on Harmonization Good Clinical Practices, the Declaration of Helsinki and regulatory requirements of Mali. Study protocols were reviewed and approved by institutional review boards of the University of Bamako Faculty of Medicine, the University of Maryland School of Medicine and the National Institute of Allergy and Infectious Diseases. Village ‘permission to enter’ was obtained from village chiefs, government officials and traditional healers prior to study initiation as described (22). Individual informed consent was obtained from the legal guardian of each participant prior to screening and enrollment. Consent of illiterate participants' guardians was documented by their thumbprints and by signatures of independent witnesses. The trial was funded and monitored by the National Institute of Allergy and Infectious Diseases/Division of Microbiology and Infectious Diseases.

### PBMC and sera collection

Blood was collected into sterile ethylenediaminetetraacetic acid (EDTA) tubes and Eppendorf tubes on admission and before antimalarial therapy, refrigerated and processed within 2 h of acquisition. Peripheral blood mononuclear cells (PBMC) were processed by density centrifugation using lymphocyte separation medium (ICN Biomedical Inc. Aurora, OH, USA) following standard techniques. PBMC were linear-rate frozen using isopropyl alcohol containers to −70°C at the field site before transferring to liquid nitrogen storage containers for transportation to the University of Maryland. HLA typing was performed sequentially at the University of Maryland and at Georgetown University. Similarly, sera were processed, frozen and transported to the University of Maryland for cytokine analysis as previously described (17).

### HLA typing: identification of alleles at the HLA-A, -C, -B and -DRB1 loci

Genomic DNA was obtained using the QIAamp 96 DNA blood kit (Qiagen, Valencia, CA) for each individual. HLA-A, -B, -C and -DRB1 alleles were initially typed at intermediate or low-resolution level using the polymerase chain reaction (PCR) and sequence-specific oligonucleotide probe (SSOP) hybridization as described previously for class I 7, 23 and the DRB1 SSOP typing kits from Lifecodes Corporation (currently Orchid Cellmark, Princeton, NJ) for class II. Subsequently, all individuals were typed by sequence-base typing (SBT) method to identify HLA-A, -B, -C and -DRB1 alleles. For class I loci, genomic DNA was amplified by PCR using locus-specific primers (23) and the Applied Biosystems Prism Big Dye terminator chemistry sequencing (PE Applied Biosystems, Foster City, CA) was performed using sequencing primers as described previously (24). For PCR amplification, a second forward primer 5B1 (5′-GCA CCC ACC CGG ACT CAG AAT CTC CT-3′) developed by us previously was used to obtain the amplification of B*51:01:02 and B*52:01:02 alleles, and a second reverse primer 3B1-AC (25) is included to obtain the amplification of B*73:01. These two primers were included in HLA-B locus-specific primers (23) to amplify all alleles at this locus. For class II, DRB1 alleles were amplified and sequenced using the HLA-DRB High-Resolution Typing System (PE Applied Biosystems) per manufacture's protocol (ABI-Prism Big Dye Terminator Cycle Sequencing Ready Reaction Protocol). DRB1 allele groups were selected and sequenced according to low-resolution SSOP typing results. To obtain resolution and to resolve ambiguous allele combinations, additional in-house PCR-specific primers and sequencing primers were used as needed (Cao et al., unpublished). Products of sequencing reaction were identified with Applied Biosystems Model 3700 DNA analyzer (PE Applied Biosystems). Sequence interpretation was conducted using dna Sequencing Analysis software, AutoAssemblerdna Sequence Assembly software, mt Navigator software and Matchtools software (PE Applied Biosystems). In the present study, we did not test for polymorphisms outside exons 2 and 3 for class I and exon 2 for class II; therefore, some groups of alleles with sequence differences only in the other exons were not distinguished. Indistinguishable alleles were assigned as the allele with the lowest number followed by a letter ‘G’ indicating that this is a group of alleles (e.g. A*02:01:01G designates the ambiguity A*02:01:01, A*02:09, A*02:43N) as detailed in our previous study (26).

### Predicting HLA class I peptide binding

To determine whether malaria peptides could bind preferentially to closely aligned HLA subtypes that differed by minor amino acid sequence differences, a computer algorithm was used to assess HLA class I binding affinity of peptides from known *P. falciparum* proteins of interest. HLA class I peptides derived from the *P. falciparum* 3D7 CSP, LSA-1, LSA-3, merozoite surface protein-1 (MSP-1), thrombospondin-related anonymous protein (TRAP) and *P. falciparum* variant erythrocyte Membrane Protein 1 (PfEMP1) with a predicted high to medium binding affinity [500 IC50 (nM) or less] for HLA-A*30:01, HLA-A*30:02 and HLA-A*33:01 were identified with the Average Neural Networks (ANN), Stabilized Matrix Method (SMM) and Average Relative Binding (ARB) algorithms 27–29. The algorithms were accessed on the Immune Epitope Database (IEDB) and Analysis Resource website (http://tools.immuneepitope.org/main/). The amino acid sequences of the *P. falciparum* 3D7 proteins were obtained from the National Center for Biotechnology Information (NCBI): CSP (XP_001351122.1), LSA-1 (XP_001347640.1), LSA-3 (XP_001349701), MSP-1 (XP_00 1352170.1) and TRAP (XP_001350088.1). *Var* gene sequences were obtained from collaborations with the University of Edinburgh (30).

### Circulating cytokine measurements

Serum levels of IL-6 and IL-10 were determined using cytometric bead array technology (BD Biosciences, San Diego, CA) and fluorescence detection by flow cytometry. Briefly, bead populations with discrete fluorescent intensities of peridinin chlorophyll protein complex (PerCP)-Cy5.5 and coated with cytokine-specific capture antibodies were added to individual patient sera and phycoerythrin (PE)-conjugated antihuman inflammatory cytokine antibodies. Simultaneously, standards for each cytokine (0–5000 pg/ml) were likewise mixed with cytokine capture beads and PE-conjugated reagent. The vortexed mixtures were allowed to incubate for 3 days enhancing the lower limit of detection. Flow cytometric analysis was performed and analyzed by a single operator and standard curves were derived from the cytokine standards. The lower limit of detection for the various cytokines evaluated ranged from 2.5 to 10 pg/ml.

### Statistical analysis

Population analyses including calculations of gene frequencies, tests for fit to expectations under Hardy–Weinberg equilibrium (HWE) and estimations of haplotype frequencies by the expectation-maximization (EM) algorithm (31) were performed in an anonymized manner using the computer package pypop (http://www.pypop.org), which is able to handle the high levels of polymorphism characteristic of the HLA loci 32–34. Allelic frequencies were obtained by direct counting, assuming no blank frequencies.

Tests for heterogeneity between specific groups and association analyses were performed using contingency table testing and a standard chi-squared measure. Alleles that were observed four or fewer times were combined for analysis. Degrees of freedom (dof) for the chi-squared analysis were calculated from the number of alleles observed five or more times, plus the combined category, minus 1. The relative predispositional effect (RPE) method was used to identify all heterogeneity in disease risk at the primary disease gene; alleles, haplotypes or genotypes with the strongest predisposing or protective effects were sequentially removed, based on their contribution to the overall chi-squared value, from the analysis until no further heterogeneity in risk effects was seen (35).

To account for linkage disequilibrium within the HLA region, stratification by the disease-associated alleles using the conditional haplotype method (CHM) was used (36). If all HLA region genes directly involved in disease susceptibility have been identified, then the relative frequencies of alleles at the other HLA loci on high-risk haplotypes should be the same in cases and controls, similarly for neutral and protective haplotypes. While fit to these expectations does not exclude the possibility that other genes in the HLA complex are involved in disease, the lack of fit unequivocally shows that all disease-predisposing genes in the region have *not* been identified.

Analyses of differences in individual cytokine levels between clinical groups with requisite HLA alleles were performed using Mann–Whitney Rank Sum analysis for populations not normally distributed (GraphPad Prism 5, LaJolla, CA; 2007).

## Results

### Clinical

We examined the HLA results between all children with malaria infection and healthy controls (*n* = 182) without malaria infection. No allelic differences were noted between healthy volunteers and those with malarial disease, but full details of these results are to be reported elsewhere (Cao et al., manuscript in preparation). HLA determinations were also examined between 237 Malian children with severe malaria and 239 with uncomplicated malaria. The median age of children with malaria was 32 months (range 3–135). Only those children who were self-reported as Dogon were included in the analysis [192 (81%) cases of severe malaria and 201 (84%) cases of uncomplicated malaria]. Of these, 171 children with severe malaria and 188 children with uncomplicated malaria were fully typed at loci, A, B, C and DRB1 of the HLA system (21 children with severe malaria and 13 children with uncomplicated malaria were only partially typed and the results were not included in the analysis).

The distribution of HLA alleles of pediatric patients of the Dogon ethnicity with severe malarial infection was compared to the allelic distribution in children with uncomplicated malaria. Severe malaria was subdivided into the following clinical groups: (1) cerebral malaria (*n* = 71), (2) cerebral with malaria-associated anemia (*n* = 25), (3) severe noncerebral (*n* = 10) and (4) hyperparasitemia (*n* = 65). Severe malaria subgroups were compared against children of the Dogon ethnicity with uncomplicated malaria (*n* = 188). Owing to clinical and HLA similarities, groups 1 and 2 (i.e. those children with manifestations of cerebral disease) were combined (*n* = 96). Children with severe malaria but not categorized as either cerebral (groups 1 and 2) or hyperparasitemic (group 4) had a small sample size (*n* = 10) that was analyzed as a single, separate group (group 3). It was observed that the allele frequency distribution of HLA-A differed significantly (*P* = 0.016) between patients with cerebral and uncomplicated malaria, whereas there was no significant difference found for HLA-B, -C nor -DRB1 (Table 1). No significant differences were observed between those children who were hyperparasitemic or fell into the ‘other’ category (see Tables S1–S4, *Supporting Information* for frequency distributions of all the alleles at HLA-A, -B, -C and -DRB1).

**1:** Allelic distribution of HLA-A, -B, -C and DRB1 loci of children with cerebral (*N* = 96) and uncomplicated malaria (*N* = 188)

Locus	Alleles (k)	Chi-square	Dof	*P* value
HLA-A	28	26.273	13	**0.016**
HLA-B	46	14.004	14	0.45
HLA-C	25	12.671	13	0.474
HLA-DRB1	26	9.667	14	0.785

Dof, degrees of freedom; HLA, human leukocyte antigen. Bold text indicates level of significance < 0.05.

### Heterogeneity of allele frequency distributions for HLA-A in patients with cerebral and uncomplicated malaria

The analysis of the distribution of alleles in patients with cerebral and uncomplicated malaria showed that only two alleles of HLA-A were significantly different. It was observed that the alleles A*30:01 and A*33:01 had higher frequency in the group of patients with cerebral disease compared to patients with uncomplicated disease [gf = 0.2031 *vs* gf = 0.1064, odds ratio (OR) = 3.17, 95% confidence interval (CI) (1.94–5.19), *P* = 0.004 and gf = 0.0781 *vs* gf = 0.0266, OR = 4.31, 95% CI (1.89–9.84), *P* = 0.005, respectively] (Table 2). No other statistically significant differences were observed for the other HLA-A, -B, -C or -DRB1 loci (data not shown).

**2:** Gene frequencies in the Dogon population of (1) HLA-A alleles of the HLA-A30 and A33 groups in cerebral and uncomplicated malaria and (2) HLA loci previously reported in other studies as associated with resistance or susceptibility to severe malaria

Allele	Malaria category			*P* value	Odds ratio	Confidence interval
Cerebral 2*n* = 192	Uncomplicated 2*n* = 376
HLA-A					
A^*^30:01	0.2031	0.1064	0.004	3.17	1.94–5.19
A^*^30:02	0.0208	0.0372	ns		
A^*^33:01	0.0781	0.0266	0.005	4.21	1.89–9.84
A^*^33:03	0.0365	0.0585	ns		
Malaria-associated HLA (other studies)					
B^*^53:01	0.1615	0.1596	ns		
Cw^*^04:01	0.1659	0.2234	ns		
DRB1^*^08:04	0.2760	0.2261	ns		
DRB1^*^13:02	0.0417	0.0638	ns		

HLA, human leukocyte antigen; ns, not significant.

Allele A*30:02 was equally represented in cerebral (gf = 0.0208) and uncomplicated malaria (gf = 0.0372) (Table 2). The alleles A*30:01 and A*30:02 belong to the same HLA serotype (HLA-A30) (37). A row by column comparison shows that the increase in frequency of the allele A*30:01 in patients with cerebral malaria was not paralleled by an increase in frequency of the allele A*30:02 belonging to the same serotype. Structurally, the alleles A*30:01 and A*30:02 differ by four nonconservative amino acid replacements at residues 70, 76, 77 and 152 (Figure 1A). These residues point to the antigen recognition site of the HLA molecule and affect the structure of the peptide-binding pockets A, B, C, E and F. Residue 77 may be of particular importance due to its location in peptide-binding pocket F, which is an important anchor site.

**1:**
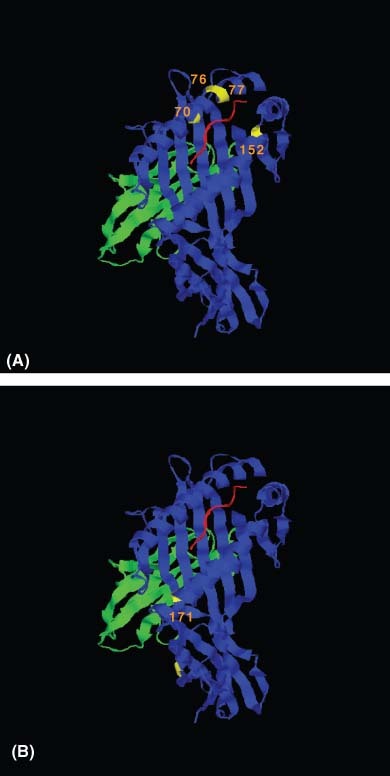
Representative ribbon diagrams depicting polymorphic positions between (A) HLA-A*30 variant molecules A*30:01 and A*30:02 and (B) HLA-A*33 variant molecules A*33:01 and A*33:03. Residue differences within the α-1 and α-2 domains of the class I molecule are depicted in yellow with positions labeled and a representative peptide (in red) lies within the peptide-binding pocket. This figure was generated in HistoCheck (http://www.mh-hannover.de/institute/transfusion/histocheck/) (13).

A similar observation was made for the alleles A*33:01 and A*33:03. The allele A*33:03 had similar frequencies in patients with cerebral (gf = 0.0365) and uncomplicated malaria (gf = 0.0585). The alleles A*33:01 and A*33:03 belong to the same serotype (HLA-A33). A row by column comparison indicates that the increase in frequency of the allele A*33:01 in patients with cerebral malaria was not paralleled by an increase in frequency in patients with uncomplicated malaria. The alleles A*33:01 and A*33:03 differ by only one nonconservative amino acid replacement at residue 171; this residue points toward pocket A of the antigen recognition site (Figure 1B).

An analysis of the distribution of the phenotypes and genotypes of patients bearing the allele A*30:01 showed that this allele was present in 37 of 96 (38.5%) patients with cerebral malaria and in 40 of 188 (21.2%) patients with uncomplicated malaria. Only two patients with cerebral malaria and none of the patients with uncomplicated malaria were homozygous of HLA-A*30:01; this difference was not statistically significant and genotypic distributions conformed to expectations under HWE.

The allele A*33:01 was present in 15 of 96 (15.6%) patients with cerebral malaria and in 10 of 188 patients (5.3%) with uncomplicated malaria. There were no patients homozygous of HLA-A*33:01 in either of these groups. In the cerebral malaria group, there were four patients (4.2%) carrying the genotype A*30:01, A*33:01, while there was only one patient (0.5%) with this genotype in the uncomplicated malaria group; the difference of the distribution of this genotype in the cerebral and uncomplicated malaria groups was not statistically significant. In all, 50% (*n* = 48) of the patients with cerebral malaria carried the A*30:01 or A*33:01 alleles, whereas they were detected in only 26.1% of the patients with uncomplicated malaria (*P*≤ 0.0001, relative risk = 2.84)

### Haplotypes bearing A*30:01 and A*33:01

The analysis of estimated haplotypes assigned by the EM algorithm showed that approximately 50% of the haplotypes bearing A*30:01 from patients with either uncomplicated or cerebral malaria also included the alleles B*42:01, Cw*17:01 and DRB1*08:04. The haplotype A*30:01-B*42:01-Cw* 17:01-DRB1*08:04 is the most frequent one in Dogon individuals free of malaria infection (haplotype frequency = 0.0734; Cao et al., manuscript in preparation); this haplotype had frequencies of 0.0493 and 0.1042, respectively, in these groups of patients. In contrast, the allele A*33:01 was found in multiple haplotypes, all of them present at low frequencies (data not shown). Table 2 shows the distribution of alleles of different HLA loci that associate with A*30:01 in patients with cerebral and uncomplicated malaria. These alleles include those that have been shown in previous studies to be associated with resistance or susceptibility to malaria, including B*53:01, Cw*0401, DRB1*08:04, DRB1*13:02 and DRB1*04 8–10, 13, 14. The differences in allelic frequencies of these haplotypes between clinical groups were small and did not reach statistical significance, suggesting a negligible impact of these alleles in the Malian population in conferring protection against severe malaria. None of these alleles at HLA-B, -C or -DRB1 were found to exhibit a statistically significant difference in patients with cerebral and uncomplicated malaria. The distribution of these alleles was not significantly different in patients with cerebral and uncomplicated malaria not carrying the allele A*30:01.

### HLA class I peptide binding predictions

The ANN, SMM and ARB algorithms predicted nonamers for HLA-A*30:01 and nonamers and decamers for HLA-A*30:02 and HLA-A*33:01. HLA class I *P. falciparum* 3D7-predicted peptides with a high to medium binding affinity for HLA-A*30:01 and/or HLA-A*33:01, but not HLA-A*30:02 (Table 3), were identified for each protein and used for further analysis. The majority of predicted peptides that were exclusive to HLA-A*30:01 and HLA-A*33:01 were identified with the ARB algorithm.

**3:** Analysis of predicted human leukocyte antigen (HLA) class I binding affinities from peptides derived from malaria antigens; liver stage antigens-1 and -3 (LSA-1 and LSA-3), merozoite surface protein-1 (MSP-1) and thrombospondin-related anonymous protein (TRAP)

Peptide sequence^a^	Malaria protein	ARB predicted IC50 (nM)^b^,^c^				
A^*^3001	A^*^3002	A^*^3301
ILYISFYFI	LSA-1	218.2	6931.9	469.2
DVNDFQISK	LSA-1	233.6	5108.4	36.3
TYVDKKLNK	LSA-3	485	646184.9	245.1
KYKVFAAPF	LSA-3	172.3	4765.5	307.3
TFSLFSSCV	LSA-3	234.9	4165.5	366.3
YSFVFDIFK	LSA-3	140.8	6909.9	412.4
LKKLVFGYR	MSP-1	143.2^b^	32917.3	82.1
SSRSNTLPR	MSP-1	35.2	1333.6	483.2
DAKSYADLK	MSP-1	82.8	7542.5	432.3
HTKEKINEK	MSP-1	14.8	1096.7	345.4
DNKERKIFI	MSP-1	174.9	110734.1	199.1
RYNNKFSSS	MSP-1	172.8	23225	226.9
DLRKIELFL	MSP-1	295.2	113974.1	214.2
LTKSYICHK	MSP-1	8.1	5802.1	438.4
HLFFELYQK	MSP-1	64.5	10847	49.7
QNFSVFFNK	MSP-1	191.3	41997.9	356
NFSVFFNKK	MSP-1	277.2	173836.5	324.1
DILNSRLKK	MSP-1	158.8	124894.4	218.9
SYKYIKESV	MSP-1	339.4	31648.8	243.7
QVRKHLNDR	TRAP	227.1	341325.6	344.4
RYIPYSPLS	TRAP	162.9	149577.9	155.6

ARB, Average Relative Binding.

a Peptides with a predicted high to medium binding affinity [500 IC50 (nM) or less] for HLA-A^*^30:01 and HLA-A^*^33:01, but not to HLA-A^*^30:02, are depicted. Results are shown for the ARB computer algorithm data unless otherwise noted.

b IC50 (nM) predicted with SMM algorithm.

c High to medium binding affinity is defined as ≤500 IC50 (nM). Low-affinity binding is represented by >500 IC50 (nM).

### Cytokine measurements

Serum cytokines (IL-6 and IL-10), which have previously been shown to be elevated in children with cerebral disease compared to children with alternate severe malaria diagnoses (17), were examined. Children with HLA alleles A*30:01 and A*33:01 and cerebral disease were compared to children with alternate alleles and cerebral disease. Children with concomitant cerebral disease and severe anemia were included in the analysis (*n* = 96). Sera were available from 46 of 48 children with HLA alleles A*30:01 and/or A*33:01 and 48 children with alternate alleles and cerebral disease (of note, 6 children were either homozygous for A*3001 or expressed both A*30:01 and A*33:01). No significant differences were noted in median IL-6 [689 (range: 10.0–10,580) *vs* 510.3 pg/ml (range: 33.1–12,509), *P* = 0.19]; however, a significant elevation in median IL-10 was noted between cerebral malaria patients bearing the A*30:01 and A*33:01 alleles (*n* = 46) as compared to cerebral malaria patients with other alleles (*n* = 48) [1727.3 pg/ml (range: 10.0–18,871) *vs* 673.2 pg/ml (range: 101.5–7631), *P* = 0.026].

## Discussion

We performed high-resolution typing of HLA class I and II loci and compared the distributions of alleles of HLA-A, -B, -C and -DRB1 loci to test for associations of HLA alleles with malaria disease severity in Malian children of Dogon ethnicity. The strong selection force that malaria exerts on the human genome coupled with the genetic diversity noted in Africa suggests that regional differences may develop that contribute to variable immune response to disease. We did not find specific alleles to be broadly associated with malaria disease but we did find allelic disparities within subcategories of malaria disease. In a large study cohort controlled for residence and ethnicity, we have found an association with the alleles A*30:01 and A*33:01 and the development of cerebral disease as compared to a group of age-matched children with uncomplicated malaria, while we did not detect associations with either the well-known allele HLA-B53 (B*53:01) or with the HLA class II block DRB1*13:02-DQB1*05:01 with severe malaria or malaria subtypes. In all, 50% of patients with cerebral malaria carried the A*30:01 or A*33:01 alleles, whereas they were detected in only 26.1% of children with uncomplicated malaria.

A*30:01 and A*33:01 are common HLA alleles in populations of African descent. The concept of HLA class I superfamilies has been defined based on similar peptide-binding motifs, structural similarities in antigen-binding grooves and cross-reactive peptide binding. HLA A*33:01 along with the alleles A3, A11, A31 and A*68:01 have all been assigned to the A3 supertype (38). A*30:01 has alternately been assigned to the A1, A3 and A24 superfamily; however, the specificity has recently been suggested to most closely resemble the HLA-A3 supertype (39). Both the A*30:01 and A*33:01 alleles have similar amino acid sequences within peptide-binding pockets and have identical sequences at amino acid 77, which is located within an important anchor site, peptide-binding pocket F. HLA-A*33:01 has been linked to persistent hepatitis B infection (40); however, no association has been noted between malaria and these two alleles. No other allele of the HLA-A3 supertype showed any statistically significant difference between the patient groups (see Tables, *Supporting Information*).

For the A*30:01 and A*33:01 alleles to contribute to susceptibility of cerebral manifestations of malaria, peptides must be able to bind differentially to these specific alleles. By examining the genetic frequency of closely related alleles such as A*30:02 and A*33:03, which have the same allelic lineage and identical HLA serotypes as A*30:01 and A*33:01 and only differ structurally by four amino acids and one amino acid, respectively, within the peptide-binding pockets, we detect essentially equal distribution of these two comparison alleles in the same children with cerebral malaria and with uncomplicated malaria. This suggests that the very small structural changes within the antigen recognition sites in the alleles of interest may result in altered binding patterns that lead to susceptibility to cerebral manifestations of malaria upon acquisition of malaria. Of note, A*30:02 does differ from A*30:01 at amino acid 77 (Asn *vs* Asp) (Figure 1).

To establish a proof of principle for this concept, it would be necessary to establish that malaria peptides restricted by particular alleles of interest would have binding patterns that differed from those of closely related alleles. As a practical measure, we could not perform this analysis using PBMC and synthesized peptides. With advanced computer algorithms, however, we could screen HLA class I-restricted nonamers and decamers within malaria antigens of interest such as LSA-1, LSA-3, MSP-1 and TRAP. The ANN, SMM and ARB algorithms have been shown to be accurate computer models enabling prediction of peptides that bind to common MHC molecules to identify T-cell epitopes 27–29. Each model has been trained on different sets of quantitative peptide binding data, has different input and output methods and may vary in utility for smaller datasets. As each model possesses unique strengths and weaknesses, we opted to examine all three predictive algorithms, although we found the best results with the ARB model. We were able to detect predicted peptides with strong binding affinity for HLA-A*30:01 and HLA-A*33:01 but not HLA-A*30:02. We did not find evidence of differential HLA binding with the malaria antigen, CSP or with *P. falciparum* variant erythrocyte surface antigens known as PfEMP1. None of the algorithms allowed for predictions using the comparator allele HLA-A*33:03. In addition, it is unclear whether peptide polymorphisms might lead to cross-strain variability between 3D7 strain high-affinity binding motifs for A*30:01 and A*33:01 and those of other malaria strains. To our knowledge, none of the proteins are known to have sequences correlating with susceptibility to cerebral malaria except for PfEMP1, which is known to precipitate adhesion and immunomodulation (41), and have been shown to display differential *var* gene transcription in Malian Dogon children with cerebral malaria as compared to alternate types of malaria. However, no allele binding motifs could be established, by computer modeling, within group A *var* genes in this study. In summary, the data suggest that very few amino acid changes can, in a computer-prediction model, profoundly influence the binding capacity within closely related HLA alleles. We realize that this computer modeling may not necessarily reflect *in vivo* HLA binding but it does provide proof of principle that differential binding is possible and should be pursued in future analyses. We speculate that this may result in altered immune responses that could influence the manifestations of malaria after infection. We hypothesize that HLA does not necessarily play a role in preventing infection, but rather appears to play a significant role in determining the disease course.

There is some evidence to suggest that activated CD8^+^ T cells may play a role in the development of cerebral malaria. Rodent models of infection with *Plasmodium berghei* show that brain-sequestered CD8^+^αβ T cells do contribute to experimental cerebral malaria 42, 43 and depletion of these cells diminishes this manifestation 43, 44. While leukocyte adhesion and sequestration has been noted at autopsy in the brains of children who died of cerebral malaria, no direct evidence that CD8^+^ T cells contribute to human cerebral malaria has been noted. The finding of an association of defined class I alleles with cerebral malaria raises the possibility that CD8^+^ T cells may be involved in the pathogenesis of this manifestation of severe malaria. The logical next step would be to examine cellular immune responses to synthesized peptides corresponding to the computer-generated peptides of interest and to determine if altered immune responses occurred in individuals bearing the HLA alleles associated to susceptibility to cerebral malaria in comparison to individuals with other alleles. Prospective studies might be prohibitively complex and involve large sample sizes. However, we were able to examine data on cytokine production in these same individuals to establish whether alterations in cytokine secretion occurred. We previously examined a large panel of inflammatory cytokines and determined that IL-6 and IL-10 levels corresponded to both severity of malaria and cerebral malaria (17). A significant elevation in IL-10 was noted between patients bearing the A*30:01 and A*33:01 alleles as compared to patients with other alleles suggesting alterations in this anti-inflammatory mediator in this group of children. No differences were noted in IL-6. Indirect evidence points to a role for IL-10 in children with cerebral malaria. As part of the inflammatory cascade, IL-10 is thought to have a suppressive effect on tumor necrosis factor-α (TNF-α), resulting in diminished malaria disease severity (45). However, IL-10 has also been shown to induce hemoxygenase-1 (HO-1), an inflammatory mediator in severe malaria (46), detected on immunohistochemical stain at autopsy in the brains of children with cerebral malaria and sequestration (47). Thus, the role of IL-10 in severe malaria remains undefined. Elevated IL-10 could merely be an anti-inflammatory mediator secreted in response to enhanced proinflammatory cytokines released in severe disease.

Our study did not identify protective effects of certain HLA alleles against severe malaria that were described previously 8–10. These differences could relate to geographic differences and strain variability of *P. falciparum*, to other host genetic differences that influence the manifestations of malaria or to distinct patterns of malaria epidemiology and transmission intensity, which in turn affect acquired immunity and disease progression. In addition, analysis may be subject to random fluctuations in allelic frequencies in a sample size of this nature; therefore, sampling error could skew results. Nevertheless, this data adds evidence that points to MHC-restricted CD8^+^ T-cell-mediated responses as being crucial in mediating immunity to malaria 48, 49. While the work from Hill et al. did not examine the subtypes of HLA-A30, the serotype HLA-B42 is present in 35%–83% of West African haplotypes bearing A*30:01 (8), suggesting that one could use HLA-B42 as an indicator in the presence of A*30:01 in a given population. The frequency of HLA-B42 as reported by Hill is much lower in the Gambia (3.5%–6.3%) as compared to Mali (22.8%–32.3%). One could therefore speculate that A*30:01 and A*33:01 may have lower frequency in the Gambia. There may be uncharacterized selection forces that result in lower haplotypic frequencies of the A30 group such that susceptibility to cerebral malaria is not seen. Whereas class I B*53:01, which has been shown to be protective in the acquisition of severe malaria in the Gambia (8), was also common in the Malian population, no such protective effect was noted between Malian children with severe malaria and either children with uncomplicated malaria or healthy controls (data not shown).

HLA class II antigens may play a direct role in parasite clearance by mediating antibody formation. Malaria-associated severe anemia, which has been associated with ongoing, recurrent infection and failure to eradicate parasitemia, is relatively uncommon in Mali, possibly related to the sharply seasonal pattern of transmission. The HLA class II haplotype, DRB1*13:02-DQB1*05:01, was shown to correlate with protection from severe disease in the Gambia (8). Interestingly, this population was noted to have a significant amount of malaria-associated severe anemia, providing a selection force for individuals with a protective class II allele. The overall prevalence of DRB1*13:02 is quite low in the Malian population (∼6.3%). Given that the DQB1*05:01 allele represents only a small fraction (data not shown) of the already low frequency of DRB1*13:02 in these populations, we were unable to discern any effect of this allele upon disease manifestations. Whether the low prevalence is related to the low frequency of malaria-associated severe anemia is unknown.

In this study, we found evidence of a possible correlation between two class I HLA alleles, A*30:01 and A*33:01, and the development of cerebral disease in a large cohort of Dogon children from Mali. We have established that a few amino acid changes within defined alleles can result in altered peptide binding in computer-prediction models. Moreover, we have shown a tangible immunologic effect (i.e. elevated IL-10 levels) that is seen among children with the alleles of interest as compared to other children with cerebral malaria. The regional differences that have been noted with respect to haplotypic variance and susceptibility or protection against manifestations of malaria could pose obstacles to the development of malaria vaccines aimed at eliciting CMI but speak to the remarkable plasticity of the human immunologic response and the effect that selective pressures play upon the diversity of the human genome.

## Supporting Information

The following supporting information is available for this article

Table S1. Frequencies of human leukocyte antigen-A (HLA-A) alleles in Dogon children with mild malaria and subgroups of severe malaria as well as matched healthy controls. Table S2. Frequencies of human leukocyte antigen-B (HLA-B) alleles in Dogon children with mild malaria and subgroups of severe malaria as well as matched healthy controls. Table S3. Frequencies of human leukocyte antigen-C (HLA-C) alleles in Dogon children with mild malaria and subgroups of severe malaria as well as matched healthy controls. Table S4. Frequencies of human leukocyte antigen (HLA)-DRB1 alleles in Dogon children with mild malaria and subgroups of severe malaria as well as matched healthy controls.

## References

[1] Kwiatkowski DP (2005). How malaria has affected the human genome and what human genetics can teach us about malaria. Am J Hum Genet.

[2] Hedrick P (2004). Estimation of relative fitnesses from relative risk data and the predicted future of haemoglobin alleles S and C. J Evol Biol.

[3] Agarwal A, Guindo A, Cissoko Y (2000). Hemoglobin C associated with protection from severe malaria in the Dogon of Mali, a West African population with a low prevalence of hemoglobin S. Blood.

[4] Modiano G, Morpurgo G, Terrenato L (1991). Protection against malaria morbidity: near-fixation of the alpha-thalassemia gene in a Nepalese population. Am J Hum Genet.

[5] Miller LH, Mason SJ, Clyde DF, McGinniss MH (1976). The resistance factor to *Plasmodium vivax* in blacks. The Duffy-blood-group genotype, FyFy. N Engl J Med.

[6] Bienzle U, Ayeni O, Lucas AO, Luzzatto L (1972). Glucose-6-phosphate dehydrogenase and malaria. Greater resistance of females heterozygous for enzyme deficiency and of males with non-deficient variant. Lancet.

[7] Cao K, Hollenbach J, Shi X, Shi W, Chopek M, Fernandez-Vina MA (2001). Analysis of the frequencies of HLA-A, B, and C alleles and haplotypes in the five major ethnic groups of the United States reveals high levels of diversity in these loci and contrasting distribution patterns in these populations. Hum Immunol.

[8] Hill AV, Allsopp CE, Kwiatkowski D (1991). Common west African HLA antigens are associated with protection from severe malaria. Nature.

[9] Hill AV, Elvin J, Willis AC (1992). Molecular analysis of the association of HLA-B53 and resistance to severe malaria [see comments]. Nature.

[10] Aidoo M, Lalvani A, Gilbert SC (2000). Cytotoxic T-lymphocyte epitopes for HLA-B53 and other HLA types in the malaria vaccine candidate liver-stage antigen 3. Infect Immun.

[11] Bouharoun-Tayoun H, Druilhe P (1992). *Plasmodium falciparum* malaria: evidence for an isotype imbalance which may be responsible for delayed acquisition of protective immunity. Infect Immun.

[12] May J, Lell B, Luty AJ, Meyer CG, Kremsner PG (2001). HLA-DQB1*0501-restricted Th1 type immune responses to *Plasmodium falciparum* liver stage antigen 1 protect against malaria anemia and reinfections. J Infect Dis.

[13] Hananantachai H, Patarapotikul J, Ohashi J, Naka I, Looareesuwan S, Tokunaga K (2005). Polymorphisms of the HLA-B and HLA-DRB1 genes in Thai malaria patients. Jpn J Infect Dis.

[14] Busson M, Vu TA, Labelle P (2002). HLA-DRB1 and DQB1 allele distribution in the Muong population exposed to malaria in Vietnam. Tissue Antigens.

[15] Osafo-Addo AD, Koram KA, Oduro AR, Wilson M, Hodgson A, Rogers WO (2008). HLA-DRB1*04 allele is associated with severe malaria in northern Ghana. Am J Trop Med Hyg.

[16] Ovsyannikova IG, Jacobson RM, Ryan JE, Dhiman N, Vierkant RA, Poland GA (2007). Relationship between HLA polymorphisms and gamma interferon and interleukin-10 cytokine production in healthy individuals after rubella vaccination. Clin Vaccine Immunol.

[17] Lyke KE, Burges R, Cissoko Y (2004). Serum levels of the proinflammatory cytokines interleukin-1 beta (IL-1beta), IL-6, IL-8, IL-10, tumor necrosis factor alpha, and IL-12(p70) in Malian children with severe *Plasmodium falciparum* malaria and matched uncomplicated malaria or healthy controls. Infect Immun.

[18] Coulibaly D, Diallo DA, Thera MA (2002). Impact of preseason treatment on incidence of falciparum malaria and parasite density at a site for testing malaria vaccines in Bandiagara, Mali. Am J Trop Med Hyg.

[19] Lyke KE, Dicko A, Kone A (2004). Incidence of severe *Plasmodium falciparum* malaria as a primary endpoint for vaccine efficacy trials in Bandiagara, Mali. Vaccine.

[20] Lyke KE, Diallo DA, Dicko A (2003). Association of intraleukocytic *Plasmodium falciparum* malaria pigment with disease severity, clinical manifestations, and prognosis in severe malaria. Am J Trop Med Hyg.

[21] Lyke KE, Burges RB, Cissoko Y (2005). HLA-A2 supertype-restricted cell-mediated immunity by peripheral blood mononuclear cells derived from Malian children with severe or uncomplicated *Plasmodium falciparum* malaria and healthy controls. Infect Immun.

[22] Diallo DA, Doumbo OK, Plowe CV, Wellems TE, Emanuel EJ, Hurst SA (2005). Community permission for medical research in developing countries. Clin Infect Dis.

[23] Cao K, Chopek M, Fernandez-Vina MA (1999). High and intermediate resolution DNA typing systems for class I HLA-A, B, C genes by hybridization with sequence-specific oligonucleotide probes (SSOP). Rev Immunogenet.

[24] Tu B, Mack SJ, Lazaro A (2007). HLA-A, -B, -C, -DRB1 allele and haplotype frequencies in an African American population. Tissue Antigens.

[25] Middleton D (1998). HLA-A and -B typing by SSOP. Newsletter, European Federation for Immunogenetics.

[26] Cao K, Moormann AM, Lyke KE (2004). Differentiation between African populations is evidenced by the diversity of alleles and haplotypes of HLA class I loci. Tissue Antigens.

[27] Nielsen M, Lundegaard C, Worning P (2003). Reliable prediction of T-cell epitopes using neural networks with novel sequence representations. Protein Sci.

[28] Bui HH, Sidney J, Peters B (2005). Automated generation and evaluation of specific MHC binding predictive tools: ARB matrix applications. Immunogenetics.

[29] Peters B, Sette A (2005). Generating quantitative models describing the sequence specificity of biological processes with the stabilized matrix method. BMC Bioinformatics.

[30] Kyriacou HM, Stone GN, Challis RJ (2006). Differential var gene transcription in *Plasmodium falciparum* isolates from patients with cerebral malaria compared to hyperparasitaemia. Mol Biochem Parasitol.

[31] Dempster AP, Laird NM, Rubin DB (1977). Maximum likelihood from incomplete data via the EM algorithm. J Royal Stat Soc.

[32] Lancaster A, Nelson M, Single R, Meyer D, Thomson G (2003). PyPop: a software framework for population genomics: analyzing large-scale multi-locus genotype data. Pac Symp Biocomp (Pacific Symposium Biocomputing).

[33] Lancaster A, Nelson M, Single R, Meyer D (2006). Software framework for the biostatistics core.

[34] Single R, Meyer D, Thomson G, Hansen JA (2006). Statistical methods for analysis of population genetic data. Immunobiology of the Human MHC. Proceedings of the 13th International Histocompatibility Workshop and Conference, Vol.1.

[35] Payami H, Joe S, Farid NR (1989). Relative predispositional effects (RPEs) of marker alleles with disease: HLA-DR alleles and Graves disease. Am J Hum Genet.

[36] Thomson G, Valdes AM (2007). Conditional genotype analysis: detecting secondary disease loci in linkage disequilibrium with a primary disease locus. BMC Proceedings.

[37] Krausa P, Munz C, Keilholz W (2000). Definition of peptide binding motifs amongst the HLA-A*30 allelic group. Tissue Antigens.

[38] Sidney J, Grey HM, Southwood S (1996). Definition of an HLA-A3-like supermotif demonstrates the overlapping peptide-binding repertoires of common HLA molecules. Hum Immunol.

[39] Lamberth K, Roder G, Harndahl M (2008). The peptide-binding specificity of HLA-A*3001 demonstrates membership of the HLA-A3 supertype. Immunogenetics.

[40] Ramezani A, Aghakhani A, Kalantar E, Banifazl M, Eslamifar A, Velayati AA (2009). HLA-A *3303* and *3301 predispose patients to persistent hepatitis B infection. J Gastrointestin Liver Dis.

[41] Miller LH, Baruch DI, Marsh K, Doumbo OK (2002). The pathogenic basis of malaria. Nature.

[42] Imai Y, Kamiyama T (1994). T lymphocyte-dependent development of cerebral symptoms in WM/Ms rats infected with *Plasmodium berghei*. Ann Trop Med Parasitol.

[43] Belnoue E, Kayibanda M, Vigario AM (2002). On the pathogenic role of brain-sequestered alphabeta CD8+ T cells in experimental cerebral malaria. J Immunol.

[44] Hermsen C, van de WT, Mommers E, Sauerwein R, Eling W (1997). Depletion of CD4+ or CD8+ T-cells prevents *Plasmodium berghei* induced cerebral malaria in end-stage disease. Parasitology.

[45] Ho M, Sexton MM, Tongtawe P, Looareesuwan S, Suntharasamai P, Webster HK (1995). Interleukin-10 inhibits tumor necrosis factor production but not antigen-specific lymphoproliferation in acute *Plasmodium falciparum* malaria. J Infect Dis.

[46] Lee TS, Chau LY (2002). Heme oxygenase-1 mediates the anti-inflammatory effect of interleukin-10 in mice. Nat Med.

[47] Clark IA, Awburn MM, Harper CG, Liomba NG, Molyneux ME (2003). Induction of HO-1 in tissue macrophages and monocytes in fatal falciparum malaria and sepsis. Malar J.

[48] Hoffman SL, Oster CN, Mason C (1989). Human lymphocyte proliferative response to a sporozoite T cell epitope correlates with resistance to falciparum malaria. J Immunol.

[49] Weiss WR, Mellouk S, Houghten RA (1990). Cytotoxic T cells recognize a peptide from the circumsporozoite protein on malaria-infected hepatocytes. J Exp Med.

