# Supplementary material for: Association of HLA alleles with Plasmodium falciparum severity in Malian children
Source: Tissue Antigens. 2011 Jun;77(6):562–71. doi: 10.1111/j.1399-0039.2011.01661.x (PMC3152196; doi:10.1111/j.1399-0039.2011.01661.x)
Supplement: Supplementary file 1 — Table S1. Frequencies of human leukocyte antigen-A (HLA-A) alleles in Dogon children with mild malaria and subgroups of severe malaria as well as matched healthy controls. Table S2. Frequencies of human leukocyte antigen-B (HLA-B) alleles in Dogon children with mild malaria and subgroups of severe malaria as well as matched healthy controls. Table S3. Frequencies of human leukocyte antigen-C (HLA-C) alleles in Dogon children with mild malaria and subgroups of severe malaria as well as matched healthy controls. Table S4. Frequencies of human leukocyte antigen (HLA)-DRB1 alleles in Dogon children with mild malaria and subgroups of severe malaria as well as matched healthy controls. [file tan0077-0562-SD1.doc]

Supplementary Table 1: Frequencies of HLA-A alleles in Dogon children with mild malaria and sub-groups of severe malaria as well as matched healthy controls

*Freq refers to frequency
**Mild refers to uncomplicated malaria 
Supplemental Table 2:  Frequencies of HLA-B alleles in Dogon children with mild malaria and sub-groups of severe malaria as well as matched healthy controls
Allele	Uninfected	Mild**	Cerebral	Cerebral+Anemia	All Cerebral	Hyperparasitemia	Severe Other	
HLA-B	2n =366	2n =376	2n = 142	2n = 50	2n = 192	2n = 130	2n =20	
 	Freq*	Counts	Freq*	Counts	Freq*	Counts	Freq*	Counts	Freq*	Counts	Freq*	Counts	Freq*	Counts	
07:02:01	0.0683	25	0.0638	24	0.0423	6	0.1000	5	0.0573	11	0.0385	5	0.1500	3	
07:05G	0.0109	4	0.0080	3	0.0141	2	0.0200	1	0.0156	3	0.0000	0	0.0000	0	
08:01	0.0137	5	0.0160	6	0.0211	3	0.0000	0	0.0156	3	0.0462	6	0.0000	0	
13:02	0.0000	0	0.0027	1	0.0000	0	0.0000	0	0.0000	0	0.0000	0	0.0000	0	
14:01	0.0191	7	0.0106	4	0.0352	5	0.0200	1	0.0313	6	0.0154	2	0.0000	0	
14:02	0.0082	3	0.0133	5	0.0070	1	0.0200	1	0.0104	2	0.0077	1	0.0500	1	
15:02	0.0000	0	0.0027	1	0.0000	0	0.0000	0	0.0000	0	0.0000	0	0.0000	0	
15:03	0.0792	29	0.0745	28	0.0704	10	0.1000	5	0.0781	15	0.0769	10	0.0500	1	
15:09	0.0000	0	0.0027	1	0.0000	0	0.0000	0	0.0000	0	0.0000	0	0.0000	0	
15:10	0.0301	11	0.0133	5	0.0423	6	0.0000	0	0.0313	6	0.0385	5	0.1000	2	
1516	0.0191	7	0.0213	8	0.0352	5	0.0000	0	0.0260	5	0.0000	0	0.0000	0	
18:01	0.0164	6	0.0106	4	0.0000	0	0.0000	0	0.0000	0	0.0231	3	0.0000	0	
27:03	0.0055	2	0.0053	2	0.0000	0	0.0000	0	0.0000	0	0.0154	2	0.0000	0	
27:05:02G	0.0000	0	0.0053	2	0.0000	0	0.0200	1	0.0052	1	0.0000	0	0.0000	0	
35:01:01	0.0956	35	0.1516	57	0.0916	13	0.1000	5	0.0938	18	0.1308	17	0.1500	3	
35:02	0.0000	0	0.0000	0	0.0000	0	0.0000	0	0.0000	0	0.0000	0	0.0500	1	
35:NEW	0.0027	1	0.0000	0	0.0000	0	0.0000	0	0.0000	0	0.0000	0	0.0000	0	
37:01	0.0027	1	0.0000	0	0.0000	0	0.0000	0	0.0000	0	0.0000	0	0.0000	0	
38:01	0.0000	0	0.0000	0	0.0000	0	0.0000	0	0.0000	0	0.0077	1	0.0000	0	
38:02	0.0000	0	0.0000	0	0.0000	0	0.0000	0	0.0000	0	0.0077	1	0.0000	0	
39:01	0.0000	0	0.0027	1	0.0000	0	0.0000	0	0.0000	0	0.0077	1	0.0000	0	
39:02	0.0000	0	0.0027	1	0.0000	0	0.0000	0	0.0000	0	0.0000	0	0.0000	0	
39:10	0.0055	2	0.0080	3	0.0000	0	0.0000	0	0.0000	0	0.0077	1	0.0000	0	
41:02	0.0055	2	0.0000	0	0.0000	0	0.0000	0	0.0000	0	0.0077	1	0.0000	0	
42:01	0.1393	51	0.1090	41	0.1479	21	0.2200	11	0.1667	32	0.1000	13	0.0500	1	
42:02	0.0137	5	0.0080	3	0.0211	3	0.0000	0	0.0156	3	0.0077	1	0.0000	0	
44:03:01	0.0109	4	0.0080	3	0.0141	2	0.0200	1	0.0156	3	0.0077	1	0.0000	0	
44:10	0.0000	0	0.0027	1	0.0000	0	0.0000	0	0.0000	0	0.0000	0	0.0000	0	
45:01	0.0574	21	0.0532	20	0.0704	10	0.0200	1	0.0573	11	0.0692	9	0.0500	1	
49:01	0.0164	6	0.0160	6	0.0000	0	0.0200	1	0.0052	1	0.0154	2	0.0000	0	
50:01	0.0164	6	0.0133	5	0.0141	2	0.0000	0	0.0104	2	0.0385	5	0.0000	0	
50:02	0.0000	0	0.0000	0	0.0070	1	0.0000	0	0.0052	1	0.0000	0	0.0000	0	
51:01:01	0.0273	10	0.0213	8	0.0282	4	0.0200	1	0.0260	5	0.0231	3	0.0000	0	
51:09	0.0000	0	0.0053	2	0.0000	0	0.0000	0	0.0000	0	0.0000	0	0.0000	0	
52:01:02	0.0765	28	0.1011	38	0.1197	17	0.0200	1	0.0938	18	0.0846	11	0.1500	3	
53:01	0.1749	64	0.1596	60	0.1479	21	0.2200	11	0.1667	32	0.1462	19	0.1500	3	
55:04	0.0000	0	0.0000	0	0.0070	1	0.0000	0	0.0052	1	0.0000	0	0.0000	0	
56:01	0.0191	7	0.0133	5	0.0070	1	0.0200	1	0.0104	2	0.0000	0	0.0000	0	
57:01	0.0000	0	0.0027	1	0.0000	0	0.0000	0	0.0000	0	0.0000	0	0.0500	1	
57:02	0.0055	2	0.0053	2	0.0070	1	0.0000	0	0.0052	1	0.0077	1	0.0000	0	
57:03	0.0027	1	0.0053	2	0.0141	2	0.0000	0	0.0104	2	0.0077	1	0.0000	0	
57:04	0.0000	0	0.0027	1	0.0000	0	0.0000	0	0.0000	0	0.0000	0	0.0000	0	
58:01	0.0301	11	0.0186	7	0.0141	2	0.0200	1	0.0156	3	0.0231	3	0.0000	0	
78:01	0.0273	10	0.0319	12	0.0211	3	0.0000	0	0.0156	3	0.0231	3	0.0000	0	
78:02:02	0.0000	0	0.0027	1	0.0000	0	0.0400	2	0.0104	2	0.0077	1	0.0000	0	
82:01	0.0000	0	0.0053	2	0.0000	0	0.0000	0	0.0000	0	0.0077	1	0.0000	0	
Total	1.0000	366	1.0000	376	1.0000	142	1.0000	50	1.0000	192	1.0000	130	1.0000	20	
 *Freq refers to frequency
**Mild refers to uncomplicated malaria
Supplemental Table 3:  Frequencies of HLA-C alleles in Dogon children with mild malaria and sub-groups of severe malaria as well as matched healthy controls


*Freq refers to frequency
**Mild refers to uncomplicated malaria 
Supplemental Table 4:  Frequencies of HLA-DRB1 alleles in Dogon children with mild malaria and sub-groups of severe malaria as well as matched healthy controls

*Freq refers to frequency
**Mild refers to uncomplicated malaria
